# Diagnosis of Whooping Cough in Switzerland: Differentiating *Bordetella pertussis* from *Bordetella holmesii* by Polymerase Chain Reaction

**DOI:** 10.1371/journal.pone.0088936

**Published:** 2014-02-19

**Authors:** Laure F. Pittet, Stéphane Emonet, Patrice François, Eve-Julie Bonetti, Jacques Schrenzel, Melanie Hug, Martin Altwegg, Claire-Anne Siegrist, Klara M. Posfay-Barbe

**Affiliations:** 1 Department of Paediatrics, Division of General Paediatrics, Children's Hospital, University Hospitals of Geneva, Geneva, Switzerland; 2 Laboratory of Bacteriology, Department of Genetics and Laboratory Medicine, Department of Medical Specialties, University Hospitals of Geneva, University of Geneva, Geneva, Switzerland; 3 Genomic Research Laboratory, Department of Genetics and Laboratory Medicine, Department of Medical Specialties, University Hospitals of Geneva, University of Geneva, Geneva, Switzerland; 4 Bioanalytica AG, Luzern, Switzerland; 5 Centre for Vaccinology and Neonatal Immunology, Departments of Pathology-Immunology and Paediatrics, University of Geneva, Geneva, Switzerland; University of Hong Kong, Hong Kong

## Abstract

*Bordetella holmesii*, an emerging pathogen, can be misidentified as *Bordetella pertussis* by routine polymerase chain reaction (PCR). In some reports, up to 29% of the patients diagnosed with pertussis have in fact *B. holmesii* infection and invasive, non-respiratory *B. holmesii* infections have been reported worldwide. This misdiagnosis undermines the knowledge of pertussis' epidemiology, and may lead to misconceptions on pertussis vaccine's efficacy. Recently, the number of whooping cough cases has increased significantly in several countries. The aim of this retrospective study was to determine whether *B. holmesii* was contributing to the increase in laboratory-confirmed cases of *B. pertussis* in Switzerland. A multiplex species-specific quantitative PCR assay was performed on 196 nasopharyngeal samples from Swiss patients with PCR-confirmed *Bordetella* infection (median age: 6 years-old, minimum 21 days-old, maximum 86 years-old), formerly diagnosed as *Bordetella pertussis* (IS*481*+). No *B. holmesii* (IS*481*+, IS*1001*−, hIS*1001*+) was identified. We discuss whether laboratories should implement specific PCR to recognize different *Bordetella* species. We conclude that in Switzerland *B. holmesii* seems to be circulating less than in neighboring countries and that specific diagnostic procedures are not necessary routinely. However, as the epidemiological situation may change rapidly, periodic reevaluation is suggested.

## Introduction

Pertussis outbreaks are reported worldwide and *Bordetella pertussis* is detected by polymerase chain reaction (PCR) with increasing frequency in nasopharyngeal (NP) samples of symptomatic patients. Teenagers and young adults represent the main reservoir, and transmit the bacterium to the more vulnerable pediatric population. In Switzerland, clusters of pertussis must be reported to the government, and current incidence of pertussis is both high (2012: 94/100'000) and increasing [1].

Although the emerging pathogen *Bordetella holmesii* was initially described as causing bacteremia in immunodeficient patients [2], it has also been recovered in NP samples of immunocompetent individuals with pertussis-like symptoms [3]. Respiratory infection with *B. holmesii* can easily be misidentified as *B. pertussis* by PCR because both genomes contain the insertion sequence IS*481* targeted by PCR assays routinely used in most laboratories. This misdiagnosis of *B. holmesii* as *B. pertussis* is common: in a recent large quality control test, only one out of 24 European laboratories properly identified a *B. holmesii* strain [4].

Accurate identification of *B. holmesii* is important for several reasons. One concerns the pertussis vaccine: some countries - including Switzerland - have implemented or are currently implementing booster pertussis vaccine doses for teenagers and/or adults. These new recommendations were implemented in response to the increasing number of patients with pertussis-like symptoms, and a shift in the age of *B. pertussis'* reservoir. The primary aim of reducing the circulation of pertussis is to protect the at-risk population, namely the young infants. However, the pertussis vaccines -both the cellular and acellular vaccine- do not protect against *B. holmesii*, according to an animal study [5]. In humans, the estimated effectiveness of the adolescent acellular pertussis booster vaccination against *B. holmesii* during a pertussis outbreak in Ohio (USA) was 36% (95%CI: −33%; +69%), compared with 67% against *B. pertussis* (95%CI: 38%; 82%) [6]. Therefore, immunized patients are likely not protected against *B. holmesii* infection and subsequent misdiagnosing of *B. holmesii* for *B. pertussis* could falsely suggest vaccine failure. This could have a strong impact on both physicians' and patients' perception of the vaccine but also lead to inappropriate public health measures. In addition, it is unclear at this time if prophylactic antibiotic treatment of all contacts of index cases, such as in *B. pertussis* infection, is necessary for *B. holmesii* infection [7]. *B. holmesii* can - unlike *B. pertussis* - cause invasive diseases, mainly in immunocompromised patients [2], [8], but also in immunocompetent hosts [9]–[11]. A work-up and treatment should therefore be considered when encountered. Finally, macrolides - antimicrobials used to treat *B. pertussis* infections - seem to have a lower activity against *B. holmesii* [12]. *In vitro* studies report that the most effective antibiotics against *B. holmesii* are fluoroquinolones and carbapenems which are rarely used to treat pertussis [2], [13]–[14].

Respiratory infections caused by *B. holmesii* have been reported worldwide in different frequencies [3], [5], [15]–[20]. A study showed that up to 20% of French adolescents and adults with pertussis-like symptoms were in fact infected with *B. holmesii* [17]. Switzerland shares borders with France and none of the Swiss laboratories routinely identifies *B. holmesii* in NP samples. The aim of this pilot study was to retrospectively re-analyze extracted DNA from *Bordetella*-positive's NP samples for the presence of *B. holmesii* in our region, and to see whether *B. holmesii* could account for part of the recent increase in laboratory-confirmed pertussis cases in Switzerland.

## Materials and Methods

Stored DNA extracted from 196 NP specimens from patients in Switzerland identified as positive for *B. pertussis* or *B. parapertussis* by routine PCR in 2009–2012 were reanalyzed by a triplex species-specific quantitative PCR (qPCR).

For the routine test, extraction of DNA from clinical specimens was carried-out with an automated extraction system (easyMAG®, BioMérieux, Marcy-l'Etoile, France). The routine real-time quantitative duplex PCR targeted IS*481* and IS*1001* and was performed in a LightCycler 2.0 (Roche, Basel, Switzerland).

Target sequences of the species-specific triplex qPCR were IS*481*, IS*1001* and hIS*1001* (Table 1). The IS*481* target is highly sensitive for *B. pertussis*: its genome contains >50 copies of the IS*481* sequence, but it is not species-specific: it is also present in the genome of *B. holmesii* (8–10 copies), and in some animal (occasionally human) isolates of *B. bronchiseptica*. The IS*1001* target is commonly used to diagnose *B. parapertussis*, although it can also be present in some strains of *B. bronchiseptica* [21]. The hIS*1001* target was selected to identify *B. holmesii*, as previously described [22]. This multiplex qPCR hence allowed the discrimination between *B. pertussis* (IS*481*+, IS*1001*−, hIS*1001*−), *B. holmesii* (IS*481*+, IS*1001*−, hIS*1001*+), and *B. parapertussis* (IS*481*−, IS*1001*+, hIS*1001*−). Randomly selected samples were then retested by a simplex qPCR using oligonucleotides recognizing the 5′ end of the pertussis toxin target (*ptxA*), which is only present in *B. pertussis*.

**Table 1:** Insertion sequence (IS)-content used in the species-specific qPCR assay.

	IS*481*	IS*1001*	hIS*1001*	C_t_ used to determine positivity^a^	Strain used for validation
*B. pertussis*	+	−	−	<25	ATCC strains n°12742
*B. parapertussis*	−	+	−	<25	CIP107610 strain, Pasteur Institute, Paris, France
*B. holmesii*	+	−	+	<25	CIP104394 strain, Pasteur Institute, Paris, France^b^
*B. bronchiseptica*	−c	−c	−	<25	QCMD strain Glasgow

qPCR: quantitative polymerase chain reaction; B: *Bordetella*; C_t_: cycle threshold; IS: insertion sequence.

a An amount of 1 ng of DNA purified from stored samples was subjected to qPCR.

Serial dilutions of purified DNA from control isolates were used to evaluate the sensitivity of the different PCR reactions, which were approximately 7–8 bacteria/reaction.

b Kindly provided by Pr. N. Guiso.

c Human and animal isolates of *B. bronchiseptica* can sometimes contain few copies of IS*481* and/or IS*1001* [22], [29].

Purified DNA from bacterial strains used for validation of the multiplex were *B. pertussis* (ATCC strains n°12742), *B. parapertussis* (CIP107610 strain, Pasteur Institute, Paris, France), *B. bronchiseptica* (QCMD strain Glasgow), and *B. holmesii* (CIP104394 strain, Pasteur Institute, Paris, France; kindly provided by Pr. N. Guiso).

Sequences of target genes were scanned for the design of sequence-specific oligonucleotides with the PrimerExpress 2.0 software (PE Biosystems, Foster City, CA, USA) using default settings. Conditions for the amplification on the CFX-96 (Bio-Rad, Hercules, CA, USA) were as follows: t1, 15 min at 95°C; t2, 15 s at 95°C; t3, 30 sec at 60°C (t2 and t3 were repeated 40 times). The volume of the PCR mixture (Bio-Rad, Hercules, CA, USA) was 20 µl and contained primers and probes at indicated concentrations (Table 2). Default analysis parameters were used with the CFX-96 device software; the standard deviation of fluorescence values recorded from cycles 3 to 15 was multiplied by 10 to define the cycle threshold line. Cycle thresholds were derived from the intercept between this line and the signal obtained during the qPCR. A reaction was considered positive when fluorescence levels exceeded the detection threshold between cycles 15 and 30. Sensitivity levels of the qPCR were assessed by using 10-fold serial dilutions of purified genomic DNA from control isolate, over a 6 log range. The detection sensitivities of *B. pertussis* and *B. holmesii* were 7–8 bacteria/sample.

**Table 2:** Sequence and characteristics of oligonucleotides used in the qPCR assay.

Target	Primers-Probes names	Sequence 5′ to 3′	Stock concentration [µM]	Concentration [µM]	Dye Reporter
	IS*481*-109F	CCGGATGAACACCCATAAGC	100	0.2	
*B. pertussis*	IS*481*-179R	CGATCAATTGCTGGACCATTT	100	0.2	
	IS*481*-130T	TGCCCGATTGACCTTCCTACGTCGA	100	0.1	FAM
	IS*1001*-202F	CGCATCAGATAAGCGGTGAG	100	0.2	
*B. holmesii*	IS*1001*-272R	CCGTGCCAATCGGTAAAGTT	100	0.2	
	IS*1001*-holm-223T	AAGGGCTGGTTGGCCTGGAGCA	100	0.1	Texas Red
	IS*1001*parapertu-1001F	GTCCTGCGTGACGAACTCAA	100	0.2	
*B. parapertussis*	IS*1001*parapertu-1071R	TGGTTCCAGGCTTGTCTTGC	100	0.2	
	IS*1001*parapertu-1022T	CGGCTCTGGTTCTACCAAAGACCTGCC	100	0.1	Atto 700
	Toxins-187F	AACGACAATGTGCTCGACCA	100	0.2	
*Toxins*	Toxins-257R	GAGACGAAAGCGCTGTTGCT	100	0.2	
	Toxins-208T	CTGACCGGACGTTCCTGCCAGG	100	0.1	FAM

qPCR: quantitative polymerase chain reaction.

After consultation of our institutional review board (Ethic Committee of Human Research of the University Hospitals of Geneva) patients did not have to provide informed written consent given that the samples had been irreversibly anonymized and kept for quality control. The ethics committee has waived the need to approve the study once the protocol was submitted.

## Results

Of the 196 available NP samples, 194 had enough material left to be reanalyzed: 188 (97%, 95% Confidence Interval (CI): 94%–99%) were confirmed as *B. pertussis*, and 5 (3%, 95%CI: 0%–5%) as *B. parapertussis*. None contained *B. holmesii* (0%, 95%CI: 0%–1.5%). One sample was negative for all three targets. None of the positive samples showed multiple signals, meaning that there was no co-infection. These results were confirmed by the simplex qPCR targeting *ptxA*. Only *B. pertussis*, as determined by IS assay, yielded positive toxin signals. Generally the Ct of the IS signal appeared 4–8 cycles earlier than that obtained for the toxin, corresponding to the relative difference of abundance within cells. Patients had a median age of 6 years (interquartile range 3–15 years; minimum 21 days-old, maximum 86 years-old), with 52% of females (Table 3).

**Table 3:** Patient characteristics.

		Gender (F/M)	*B. pertussis* (n)	*B. parapertussis* (n)	No results (n)*
**All patients**		101 F/95 M	188 (96%)	5 (3%)	3 (2%)
**Age group**	0–9 y	63 F/50 M	107 (55%)	5 (3%)	1 (1%)
	9–17 y	19 F/22 M	39 (20%)		2 (1%)
	>17 y	19 F/23 M	42 (21%)		

F: female, M: male, n: number, y: years-old.

* One sample was negative for all tested targets whereas volume of remaining material for two other samples was not sufficient to perform the assays.

## Discussion

We detected no *B. holmesii* in NP samples from Swiss patients with PCR-confirmed *Bordetella* infection reported as positive with a routine, non-discriminating, *Bordetella* PCR assay. Therefore it seems unlikely that *B. holmesii* is responsible for the reported increasing incidence of pertussis diagnosis in Switzerland. This result is in accordance with other studies from various geographical origins: *B. holmesii* was not recovered in samples from Tunisian infants [23], or patients from Finland [24], the Netherlands [24], Canada (Alberta) [25] and Australia [26]. In contrast, *B. holmesii* was identified in 0.4%–29% of *Bordetella*-positive NP samples from Canada (Ontario) [16], in children from Argentina and Chile [15], [19], and adolescents and adults from France [17], Japan [18], and USA (Massachusetts, Ohio) [3], [5]–[6].

Our results were unexpected for several reasons. First, we recently identified *B. holmesii* in blood samples of two immunocompromised patients hospitalized in our institution and hence expected the “endemic” presence of *B. holmesii* in our population [27]. In addition, infection due to *B. holmesii* has been detected in several patients from different regions of Switzerland (Swiss laboratories network, personal communication). Hence, it is possible, that *B. holmesii* circulates in our country, but that its incidence is low or that it follows epidemic curves such as reported for *B. pertussis* [28]. Second, *B. holmesii* was reported in neighboring France [17]. Given the retrospective setting of our pilot study, we unfortunately lack specific information on the patients' clinical presentation, immunization history and socio-demographic characteristics. Therefore, it is unclear whether and how our patients differ from the French patients reported by Njamkepo et al [17]. Age difference could not explain this dissimilarity: the majority of our patients were 0–9 years-old children (113/196, 58%), but there were also 41 adolescents (9–17 years-old, 21%) and 42 adults (>17 years-old, 21%), which is comparable to the age distribution of the patients reported in the French study (119 children, 20 adolescents, 39 adults).

The highest frequency of *B. holmesii* has been described in adolescents and adults with pertussis-like symptoms. However, it is now known that *B. holmesii* can cause respiratory infections in all age groups since children - including seven <6 months-old - were reported in recent publications from Argentina and Chile [15], [19]. In our study, all age groups were represented: we were therefore surprised not to find any *B. holmesii* in patients with PCR-confirmed *Bordetella* infection. A limit in our qPCR's detection level could explain the fact that we had no positive patient. However, serial dilutions showed a sensitivity level around 8 bacteria, sufficient to identify *B. holmesii* in the specimen, even in a low amount. In all positive reactions, the fluorescent signal appeared quite early (earlier than cycle 30) reflecting a significant abundance of the amplified target gene.

Finally, little is known on *B. holmesii* epidemiology and its interaction with *B. pertussis'* epidemiology. It is possible that some other factors (genetic, pertussis immunization coverage, management following suspicion of *B. pertussis* index case, or local pertussis outbreaks) influence the transmission of *B. holmesii*, and differ between countries explaining the observed difference.

The two main limitations of this study are its retrospective aspect, as discussed earlier, and the small number of samples. It would have been interesting to have access to the patients' clinical data, and to compare them with other studies looking at signs and symptoms in patients with *Bordetella holmesii* versus non-*holmesii* infections. It is unclear at this time how to differentiate clinically patients presenting with whooping cough but infected with a different *Bordetella*. It is estimated that around 5000 pertussis cases occur per year in Switzerland [1]: we tested less than 200 samples. However, in our study all age groups are represented and the bacteria were sampled from symptomatic patients in one region sequentially.

The difference in *B. holmesii* incidence between countries [3], [5]–[6], [15]–[19], [23]–[25] remains unknown, and suggests that active surveillance should be continued. However, since no *B. holmesii* was isolated in our samples and because of the higher costs of the *Bordetella* species-specific PCR, there is currently no urge to implement specific diagnostic procedures routinely. The priority remains to diagnose *B. pertussis*. Based on our results and given the importance to prevent misdiagnosis of *B. holmesii* as *B. pertussis*, we would highly recommend to all regions facing with an increasing incidence of pertussis diagnosis to use *Bordetella* species-specific tests in order to see whether and to what extend *B. holmesii* is contributing to this raise. Depending on the result, it should than be discussed if species-specific tests are needed routinely; they might not be indicated if the prevalence of *B. holmesii* is very low, given their higher cost. However, the epidemiology of both species can change rapidly and a second study with species-specific tests should be considered a few years later. Therefore, for the moment, in our opinion, in Switzerland, specific PCR diagnosis for *B. holmesii* should only be used for epidemiological reasons, in unusual patient situations, or in vaccine studies.
